# Intraperitoneal collagenase as a novel therapeutic approach in an experimental model of colorectal peritoneal carcinomatosis

**DOI:** 10.1038/s41598-020-79721-0

**Published:** 2021-01-12

**Authors:** D. García-Olmo, P. Villarejo Campos, J. Barambio, S. Garcia Gomez-Heras, L. Vega-Clemente, S. Olmedillas-Lopez, H. Guadalajara, M. Garcia-Arranz

**Affiliations:** 1 New Therapies Laboratory, Health Research Institute-Fundación Jiménez Díaz University Hospital (IIS-FJD), Avda. Reyes Católicos, 2, 28040 Madrid, Spain; 2 Department of Surgery, Fundación Jiménez Díaz University Hospital, Avda. Reyes Católicos, 2, 28040 Madrid, Spain; 3 Department of Surgery, Universidad Autónoma de Madrid, C/Arzobispo Morcillo s/n, 28034 Madrid, Spain; 4 Department of Human Histology, Universidad Rey Juan Carlos, Avda de Atenas s/n, 28922 Alcorcón, Spain

**Keywords:** Drug development, Colorectal cancer

## Abstract

The usefulness of local collagenase in therapeutic approaches to solid tumors has been tested recently. In this study, we evaluate the safety and efficacy of intraperitoneal collagenase associated or not to mitomycin for treatment of colorectal peritoneal metastases in an experimental rat model. Using a fixed-dose procedure, we found that a dose of collagenase of 37 IU/mL administered for 15 min with a hyperthermia pump at 37.5 °C, both in isolation or associated to sequential treatment with intraperitoneal mitomycin, led to a macroscopic decrease in tumor volume as evaluated by the modified peritoneal cancer index (mPCI). Concerning the safety of the procedure, the animals showed no physiological or behavioral disorders during 8 weeks of follow-up. Local treatment for peritoneal metastases of colorectal origin with intraperitoneal collagenase has proved safe and effective in an experimental murine model. Therefore, the stroma-first approach by enzymatic breakdown of collagen from the tumor's extracellular matrix provides a new therapeutic target for colorectal peritoneal metastases.

## Introduction

The components of the tumor microenvironment are related to drug resistance in several tumors. One such component, the tumor stroma, protects against the arrival of therapeutic agents to target cancer cells^1^. However, this microenvironment may be modified by reshaping the extracellular matrix (composed mainly of collagen fibers), thereby facilitating the penetration and delivery of drugs into tumors. In this way, therapeutic approaches could achieve a higher concentration of drugs within tumor cells and improve the tumor response^2^.

Currently, local administration of collagenase is a standard treatment for benign processes such as Dupuytren’s disease, Peyronie’s disease, and enzymatic debridement in cutaneous ulcers^3–5^. Although this treatment is not yet validated for clinical use in cancer, in vivo studies using intratumoral or intravenous routes to deliver collagenase have been carried out in murine tumor models.

Intravenous dose above 500 μg (0.5%) have been shown to be lethal in murine models due to abdominal and pulmonary bleeding^2^. Meanwhile, local administration of intratumoral collagenase at a dose of 37.5 U/mL did not demonstrate toxicity in any organ and the enzyme did not locate outside the tumor^6^.

Degradation of the extracellular matrix using intravenous collagenase nanoparticles has been shown to improve drug penetration in mice bearing cancer^7^. A chamber-based approach for local collagenase application in a tumor-bearing rat model has shown that proper control of time, pressure, and enzyme concentration is essential to limit possible toxicity^8^.

To date, no reports of intraperitoneal administration of collagenase for treatment of colorectal peritoneal metastases have appeared in the literature. The aim of this study is to show the results obtained by using intraperitoneal collagenase to treat peritoneal surface carcinomatosis in an experimental rat model.

## Methods and results

### Legal and ethical considerations

The present study has been approved by the Research Ethics Committee of the University Hospital Fundación Jiménez Díaz (no. PIC/75-2016) and the Committee on Animal Research and Ethics of the University General Hospital of Albacete (no. 23-2017). All experiments were performed in adherence of national and international regulations on the protection of experimental animals. The animals were reared in accordance with Directive 2010/63/UE and maintained with unlimited access to water and standard rat chow. The environmental conditions (light, ambient temperature, and relative humidity) were kept constant.

### Step 1: Estimation of collagenase solution concentration and acute toxicity

We used a commercially available collagenase for intraperitoneal administration (LYPOSMOL BIOTECH). Irrigation of the abdominal cavity was carried out with collagenase in a physiological solution (RINGER LACTATE, Braun, Spain); collagen concentration was expressed in units/milliliters (U/mL). To optimize the enzymatic activity of collagenase, we used temperatures ranging from 36 to 37.5 °C, depending on the manufacturer's indications. Intraperitoneal collagenase and mitomycin (INIBSA, Spain) were administered with the use of a heated pump perfusion system approved for experimental and clinical use (COMBAT BRS RECIRCULATION SYSTEM, Hertfordshire, U.K.). When adjusting doses, we calculated the units of enzymatic activity (U), defined as the catalytic activity responsible for the transformation of 1 µmol of substrate per minute under optimal conditions.

When studying acute intraperitoneal toxicity, we used the fixed-dose method as an alternate means of determining the lethal dose 50; in this way, we attempted to reduce and refine experimental procedures in animals. We tested doses of 350, 175, 87.5, 70, 37, 17.5, 8.75, and 3.5 U/mL in Wistar rats with ages ranging from 6 to 8 weeks and an approximate weight of 250 mg. Peripheral blood samples were taken from each animal to quantify collagenase concentration. Histological studies were performed in all rats.

### Results

**Figure 1:**
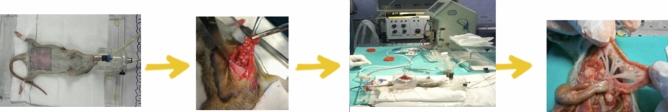
Flow-chart. Scheme of the surgical protocol followed for treatment with intraperitoneal collagenase. First step, evaluation under anesthesia of a modified Peritoneal Carcinomatosis Index (mPCI); second step, continuous perfusion system (COMBAT-BRS) was placed in the peritoneal cavity, in order to deliver collagenase or mitomycin in the designed regimen; third step, post-treatment evaluation of the mPCI.

We found that 350 U/mL was a toxic lethal dose; all animals that received this dose died within 1 h of administration, and necropsy showed that the cause of death was massive abdominal bleeding. The optimal dose of intraperitoneal collagenase was 37 U/mL, and the optimal exposure time was 15 min (Fig. 1).

When administered under these conditions, collagenase was not detected in the blood of the animals tested. Furthermore, histologic examination revealed that concentrations below 70 U/mL did not induce severe tissue damage.

### Step 2: Murine model of peritoneal carcinomatosis

When designing an appropriate experimental model of colorectal peritoneal carcinomatosis, we chose our previous model consisting of BD-IX syngeneic rats and the cell line DH/K-12 (also known as DHD/K12-TRb)^9^.

Animals: We used BD-IX syngeneic rats that were 6 weeks of age, selecting these independently of sex. To implant the tumor on the peritoneal surface, the animals were weighed and subjected to general anesthesia (75 mg/kg ketamine plus 25 mg/kg xylocaine). The tumor was implanted by means of an intraperitoneal injection of 1 × 10^6^ cells in 0.25 ml and phosphate-buffered saline. After the tumor was successfully implanted, we selected those rats with recognizable peritoneal carcinomatosis by palpating the abdominal wall on a weekly basis after the first month. Eighty percent of the rats developed palpable peritoneal carcinomatosis within 2 months of the injection.

Rats with confirmed peritoneal carcinomatosis were randomly distributed among the experimental groups in the study. Each experimental group consisted of 6 rats (the number of specimens needed was previously calculated based on the results of preliminary tests).

The control group, which received intraperitoneal collagenase without peritoneal carcinomatosis, was established in the previous step (step 1).

Four groups were established: a (Control group), receiving peritoneal carcinomatosis without treatment; B, the group receiving intraperitoneal collagenase; C, the intraperitoneal mitomycin group, and; D, Sequential intraperitoneal treatment group (collagenase followed by mitomycin treatment).

The collagenase dose used was 37 U/mL for 15 min at 37.5 °C, in line with our previous toxicity study.

Mitomycin is a chemotherapy drug that has been proven effective for the treatment of peritoneal carcinomatosis of colorectal origin. The dose of mitomycin used was 35 mg/m^2^ (usual dose for humans) for 10 min.

Animals were euthanized with an intravenous dose of sodium thiopental at 1 and 8 weeks after the end of treatment. Euthanasia was also performed when animals showed visible signs of uncontrollable suffering or pain, according to the endpoint criteria.

Outcomes were evaluated according to three variables:

1. Tumor volume reduction related to each treatment as evidenced by macroscopic examination, based on the mPCI before and after treatment.
2. The occurrence of adhesions in the abdominal cavity.
3. The Irwin Test, used to evaluate the toxicity of each group.

All animals were evaluated daily after surgery to determine their health and behavior according to a monitoring table (data not shown). To detect circulating collagenase in peripheral blood, samples were taken from each rat. An external laboratory performed ELISA tests against matrix metalloproteinase-1 and -2 (MMT1 and MMT2). An independent surgeon assessed the extension of the peritoneal metastases based on the mPCI score and the characteristics of intra-abdominal adhesions.

### Variables measurement

- Modified PCI scale:
  - More than 20 nodules equals mPCI of 3
  - Between 20 and 5 nodules equals mPCI of 2
  - Less to 5 is equivalent to mPCI of 1
- Abdominal adhesion scale:
  - no adhesion: 0
  - manually separated adhesions: 1
  - adhesions that need instruments to separate them: 2
- Irwin test scale (behaviour and autonomic disturbs)
  - Absence of disorders: 0
  - low disorders: 1
  - moderate disorders: 2
  - high disorders: 3

Before starting the experimental treatment we performed a laparotomy that allows us to assess the extent of the tumor disease in the abdominal cavity of each rat and we confirmed extensive peritoneal carcinomatosis, with a mPCI that reached score 3 in all animals.

### Statistical analysis

We applied a mixed-methods design to quantify the observations made with regard to each variable^10^. One-way ANOVA followed by Tukey’s post-hoc tests for multiple comparisons were performed. Results with a value of P < 0.05 were considered significant. Analyses were carried out using the SPSS statistical package, version 23.0, software for Windows (SPSS, Chicago, IL, USA).

### Results

The peritoneal carcinomatosis rate achieved with our model was 80%, according to previous experiences. No unexpected deaths occurred during procedures. We found no traces of collagenase in the ELISA tests performed on blood samples. All animals were euthanized after 2 months of follow-up, except for rats of the mitomycin-treated group (C); in this group, 5 rats were euthanized early due to signs of pain and discomfort that could not be controlled with analgesics (buprenorphine and tramadol according to our follow-up protocol).

The animals were autopsied and macroscopic findings on the necropsy were evaluated (Table 1).

**Table 1:** Macroscopic findings on the necropsy.

Group	Tumor volumen and systemic metastases after treatment	Adhesion characteristics	Macroscopic tissue damage
A	Numerous nodules and masses that diffusely involve peritoneal surfaces, omentum Lung metastases	Film and adhesions	No
B	Lower tumor volume in peritoneal surfaces without other affected organs Fatty tissue lysis of the omentum No distant metastases	Adhesion-free	Slight tissue damage
C	Several nodules located on peritoneal surfaces, without other organs involved No distant metastases	Dense adhesions	Retracted abdominal organs Hematic omentum Inflamed bowel without intestinal perforation
D	Massive decrease in tumor volume. Small and isolated tumor nodules that were easily removed from peritoneal surfaces Fatty tissue lysis of the omentum No distant metastases	Adhesion-free	Slight tissue damage

A or Control Group (untreated peritoneal carcinomatosis), B or Intraperitoneal Collagenase Group, C or Intraperitoneal Mitomycin Group, D or Sequential Intraperitoneal Treatment Group (first collagenase treatment followed by mitomycin treatment).

The control group (A) had numerous tumors located on peritoneal surfaces and in the lungs of the rats; no other organs were involved. The intraperitoneal collagenase group (B) showed a low tumor volume on peritoneal surfaces and no other organ involvement or fatty-tissue lysis of the omentum. In the intraperitoneal mitomycin group (C), we observed several tumor nodules, retracted abdominal organs, hematic omentum, and inflamed bowel without perforation. Finally, rats undergoing sequential intraperitoneal treatment (D) showed isolated, small tumor nodules that were easily removed from peritoneal surfaces.

We observed differences in tumor-volume reduction among groups (F: 22.2, P = 0.000). The collagenase and sequential groups exhibited greater degrees of reduction (Tukey’s post hoc test, P < 0.05), and between these two groups there were no differences (P = 0.919).

Regarding the appearance of adhesions after surgery, intergroup differences were found the occurrence of adhesions in the abdominal cavity (F: 70.6, P = 0.000). Intraabdominal adhesions were less common in the collagenase and sequential groups (Tukey’s post hoc test, P < 0.05); between these two groups there were no statistically significant differences (P = 0.999).

Regarding toxicity parameters, there were differences found among groups (F: 16.4, P = 0.000). The intraperitoneal mitomycin group had higher toxicity than other experimental groups (Tukey’s post hoc test, P < 0.05).

The analysis of variables among different groups was shown in Table 2.

**Table 2:** Statistical analysis of variables.

Rats groups	Modified PCI			Abdominal adhesions			Irwin test		
	${\overline{\text{x}}}$ ± SD	Post hoc analysis (P value)		${\overline{\text{x}}}$ ± SD	Post hoc analysis (P value )		${\overline{\text{x}}}$ ± SD	Post hoc analysis (P value )	
A	3.00 ± 0.00	B	0.000	0.60 ± 0.547	B	0.006	0.60 ± 1.341	B	0.756
		D	0.000		D	0.006		D	0.999
		C	0.163		C	0.000		C	0.000
B	1.33 ± 0.516	A	0.000	0.00 ± 0.00	A	0.006	0.166 ± 0.408	A	0.756
		D	0.919		D	0.999		D	0.634
		C	0.004		C	0.000		C	0.000
C	2.40 ± 0.547	A	0.163	2.00 ± 0.00	A	0.000	3.00 ± 0.00	A	0.000
		B	0.004		B	0.000		B	0.000
		D	0.001		D	0.000		D	0.000
D	1.16 ± 0.408	A	0.000	0.00 ± 0.00	A	0.006	0.666 ± 0.516	A	0.999
		B	0.919		B	0.999		B	0.634
		C	0.001		C	0.000		C	0.000

${\overline{\text{x}}}$ mean, *SD* standard deviation. The mean difference is significant at the 0.05 level, with a 95% confidence interval. Rats groups: A Control group; B, collagenase treatment group; C, mitomycin treatment group, and; D, collagenase followed by mitomycin treatment group.

**Figure 2:**
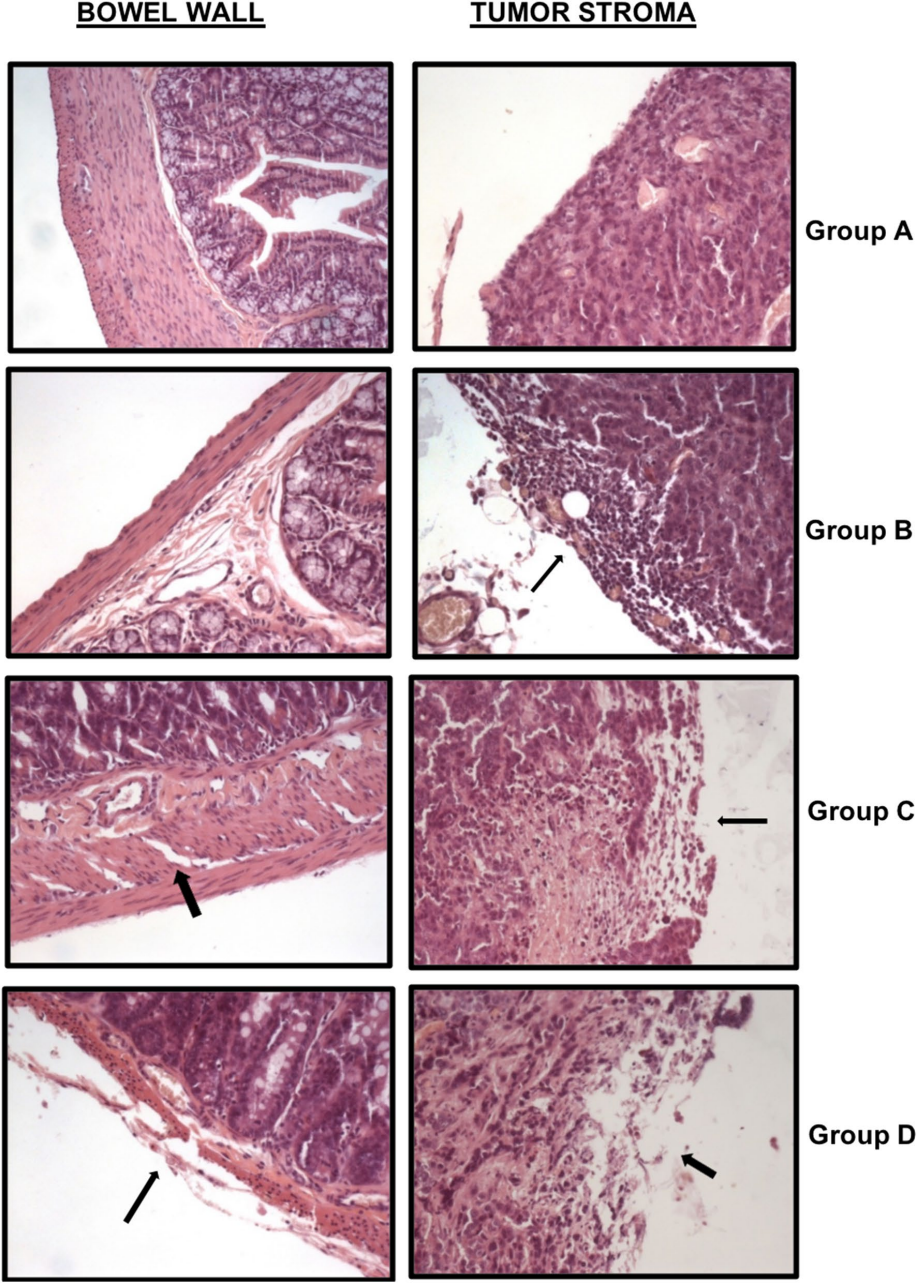
Histological intestine and tumor studies. Left: we analyzed the bowel wall in all groups: in Group B (Intraperitoneal Collagenase Group), a slight thinning of the external muscle layer in the bowel wall was present; in Group C (Intraperitoneal Mitomycin Group), the serosa layer was broken and both external and internal muscle layers were affected with this treatment.; and Group D (Sequential Intraperitoneal Treatment Group: collagenase followed by mitomycin treatment) a thinner external muscle wall and edema in the internal muscle layer existed, which caused a slight cell disorganization. Right: we analyzed the effect of treatments on peritoneal tumor implants. In Group B, the stroma and the superficial tumor area were altered. Group C, mild stroma disorganization was produced. Group D, the stroma was disaggregated and tumor cells liberated. All photographs are hematoxylin–eosin stained (×200).

Histopathologic analysis: the control group (A) had several cell implants covered with a hard stroma and highly conserved structures as previously described. Group B (Intraperitoneal Collagenase) showed less attached tumor masses and a softer stroma surrounding the tumor. No damage was observed in any other organ except the slightly thinner intestinal wall. Group C (Intraperitoneal Mitomycin) showed similar tumor masses to Group A, with hemorrhagic areas in the bowel and involvement of the muscular layers, thinner externally and with edema internally. Around the tumor masses, mild stroma disorganization and adherences in tissues were described. Finally, in Group D (Sequential Treatment), the pathologist observed less tumor masses than previous groups with microscopic hemorrhagic areas in the serosa and muscular layers of the small intestine. Around the tumors, the stromal was disaggregated and a few tumor cells were free (Fig. 2).

### Ethics approval and consent to participate

The present study was approved by the Research Ethics Committee of the University Hospital Fundación Jiménez Díaz (nº PIC/75–2016) and the Committee on Animal Research and Ethics of the University General Hospital of Albacete (Nº 23–2017). All experiments were performed following national and international regulations on the protection of experimental animals.

## Discussion

Several tumors characterized by a poor response to drugs have shown an overexpression of the extracellular matrix with abundant collagen. These fibrous tumors include cholangiocarcinomas, pancreatic cancers, sarcomas, and even peritoneal metastases^11–14^.

In addition, these cancers all exhibit poor vascularization in the tumor microenvironment and one that triggers hypoxia, which would play an important role in the tumor-reactive stroma^11,15^.

The extracellular fibrous matrix of the tumor microenvironment acts as a barrier, hindering access by chemotherapy drugs (large-size molecules) to cancer cells and thus weakening tumor response^16^.

The proteolytic activity of collagenase on the extracellular matrix could facilitate the arrival of chemotherapeutic agents to the tumor, thereby improving response to chemotherapy.

Collagenase also degrades tumor microvascular-associated collagen and reduces vascular resistance and microvascular pressure. These two phenomena reduce the transcapillary pressure gradient and facilitate drug delivery from the capillary to the tumor cells^17^.

Therefore, collagenase acts on both the extracellular matrix and tumor microvascularization, increasing the transcapillary pressure gradient, which facilitates the diffusion and concentration of drugs into the tumor.

We intuited that intraperitoneal administration of collagenase would be suitable for the treatment of peritoneal carcinomatosis, since collagenase acts directly on the collagen matrix of the tumor implants. Moreover, the rapid inactivation of collagenase when administered intravenously must be taken into account^18^.

Direct contact between collagenase and the collagen of tumor implants could be related to a decrease in the half-life of the enzyme activity and to an absence of toxicity under adjusted time and concentration conditions.

Experimental research has showed that pre-treatment with intravenous collagenase improves the concentration of drugs in fibrous tumors such as pancreatic cancer^7^.

Our results reveal that the administration of intraperitoneal collagenase is safe and effective in a murine model of peritoneal carcinomatosis. Intraperitoneal collagenase solution should be administrated by a recirculating perfusion system. The extensive distribution of peritoneal metastases makes this route ideal for achieving a complete distribution of the collagenase solution throughout the abdominal cavity. Preconditioning peritoneal surfaces with intraperitoneal collagenase may improve the action of chemotherapy drugs administered both intravenously and intraperitoneally. Furthermore, this enzymatic treatment has been shown to decrease intra-abdominal adhesions and can assist in releasing tumor masses attached to the omentum and bowel. In addition, sequential treatment could prevent tissue damage caused by mitomycin (Fig. 4). These histologically confirmed data suggest that intraperitoneal collagenase is not toxic and may also protect tissues treated with intraperitoneal chemotherapy.

In our study, we observed that high concentrations of collagenase may produce local toxicity (thinning of the muscular layer of the bowel), which makes it important to provide homogeneous parameters as concerns the concentration and length of the enzymatic treatment.

**Figure 3:**
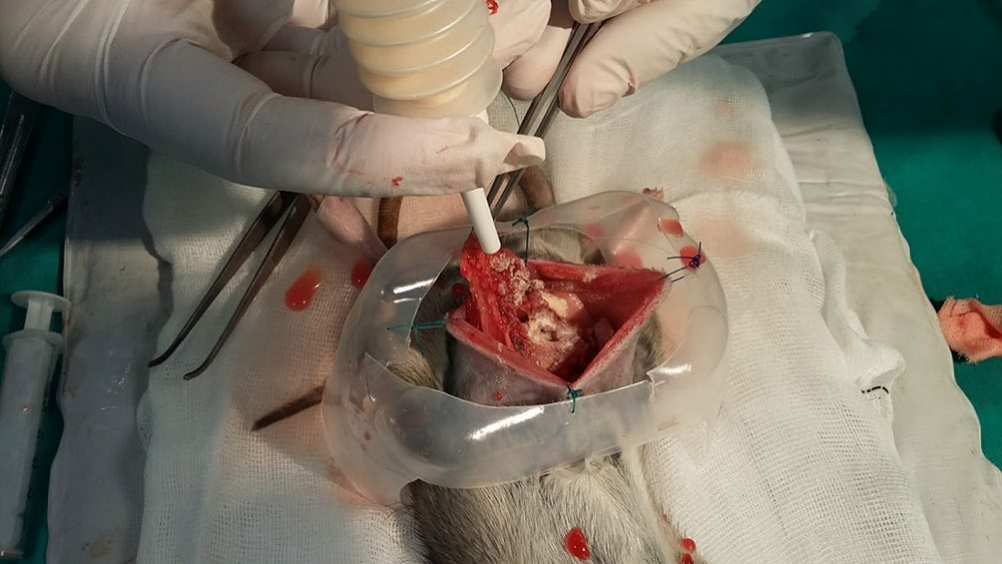
Stopping collagenase reaction. Hydrolyzed collagen was applied after treatment with collagenase and we observed a quick cessation of the enzymatic activity.

Despite the highest concentration of collagen in the tumor matrix leads to the early stopping of the enzyme activity, during our study, we decided to test how to neutralize it. Therefore, we added hydrolyzed collagen (HEMOSNOW; Biom'up, France) after collagenase perfusion and observed an immediate cessation of the reaction (Fig. 3).

**Figure 4:**
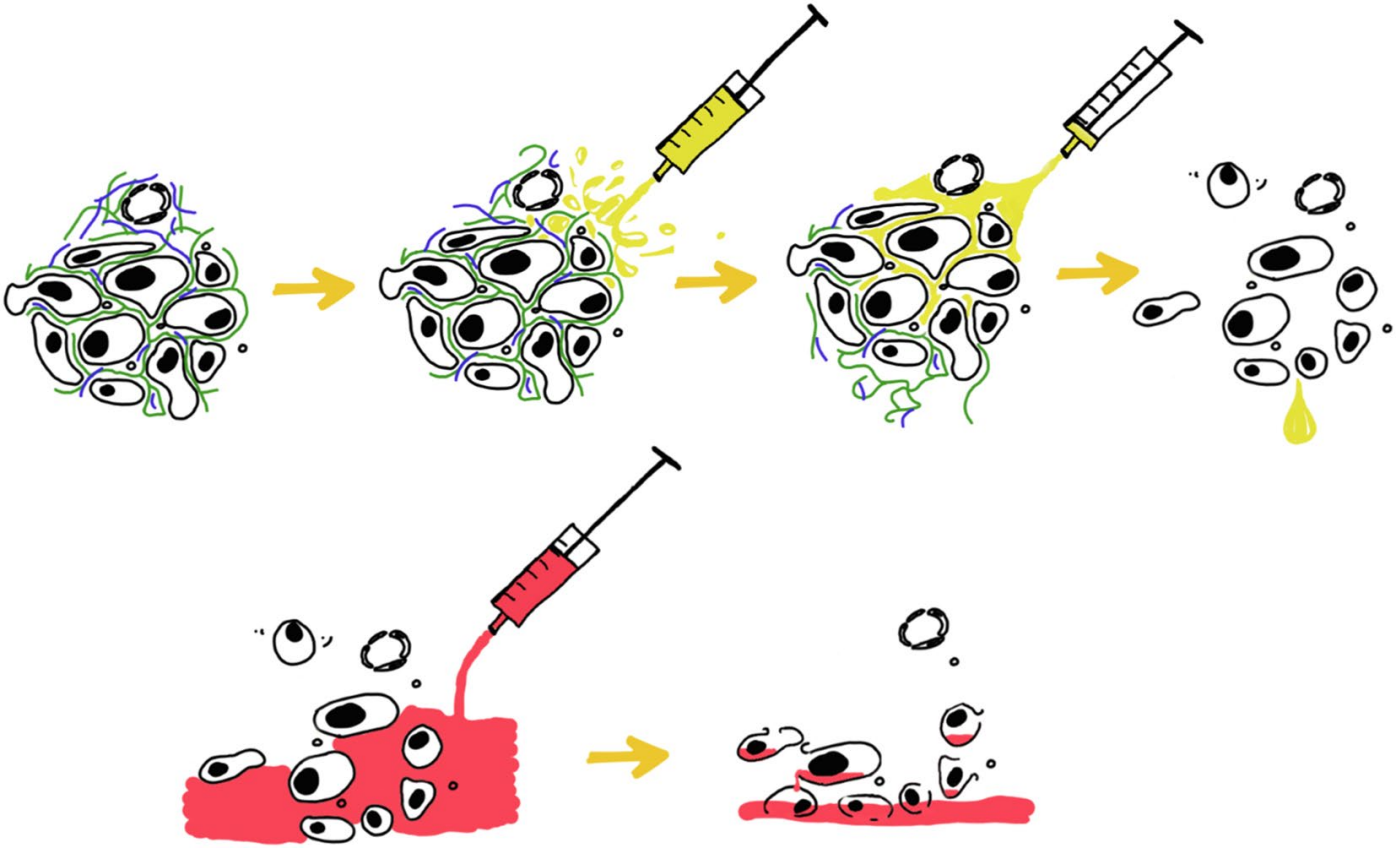
First treating the stroma. Preconditioning of peritoneal surfaces by intraperitoneal collagenase perfusion (yellow) can improve the action of chemotherapy drugs. Treating the stroma first, we released tumor cells from the stroma, making them vulnerable to chemotherapeutic agents (red).

In the future, studies should be carried out in large animals before this treatment may be tested in a clinical trial.

### Study strengths and limitations

The main limitation of our study concerns the toxicity arising from the group of rats treated with intraperitoneal mitomycin. We chose mitomycin because it is the hyperthermic intraperitoneal chemotherapy (HIPEC) drug used to treat patients with peritoneal metastases of colorectal origin. The usual dose (35 mg/m^2^) was adjusted to the body surface of each rat. Tissue damage in rats has been reported with doses of mitomycin higher than 2 mg/kg^19^.

However, this is also one of the strengths of our research, as we have proven that pre-conditioning treatment with collagenase could avoid tissue damage.

## Data Availability

The datasets analysed during the current study available from the corresponding author on reasonable request.

## Abbreviations

ANOVA: Analysis of variance
ELISA: Enzyme-linked immunosorbent assay
mg/kg: Milligram/kilogram
mg/m^2^: Milligram/square meter
mL: Millilitre
mPCI: Modified peritoneal carcinomatosis index (peritoneal cancer index adapted to this experiment)
p: P value
U: Units of enzymatic activity
U/mL: Units of enzymatic activity/milliliter
